# How Grammar Introduces Asymmetry Into Cognitive Structures: Compositional Semantics, Metaphors, and Schematological Hybrids

**DOI:** 10.3389/fpsyg.2019.02275

**Published:** 2019-10-17

**Authors:** David Gil, Yeshayahu Shen

**Affiliations:** ^1^Max Planck Institute for the Science of Human History, Jena, Germany; ^2^The Program of Cognitive Studies of Language and Its Uses, Tel Aviv University, Tel Aviv, Israel

**Keywords:** compositional semantics, metaphor, hybrid, asymmetry, thematic roles, ontogeny, phylogeny, conceptual combination

## Abstract

This paper presents a preliminary and tentative formulation of a novel empirical generalization governing the relationship between grammar and cognition across a variety of independent domains. Its point of departure is an abstract distinction between two kinds of cognitive structures: symmetric and asymmetric. While in principle any feature whatsoever has the potential for introducing asymmetry, this paper focuses on one specific feature, namely thematic-role assignment. Our main empirical finding concerns the role of language, or, more specifically, grammar, in effecting and maintaining the distinction between symmetric and asymmetric cognitive structures. Specifically, whereas symmetric structures devoid of thematic-role assignment more commonly occur in a non-grammatical and usually also non-verbal medium, asymmetric structures involving thematic-role assignment are more likely to be associated with a grammatical medium. Our work draws together three independent strands of empirical research associated with three diverse phenomenological domains: compositional semantics, metaphors and schematological hybrids. These three domains instantiate conceptual combinations, bringing together two or more subordinate entities into a single superordinate entity. For compositional semantics this consists of a juxtaposition of constituent signs to form a single more complex sign; for metaphors this entails the bringing together of two different concepts in order to produce a comparison; while for schematological hybrids this involves the combination of different entities to form a single new hybrid entity. Our empirical results reveal a remarkable parallelism between the above three domains. Within each domain, symmetric structures tend to be associated with a non-verbal or otherwise non-grammatical medium, while asymmetric structures are more frequently associated with a grammatical medium. Thus, within each domain, grammar introduces asymmetry. More specifically, we find that in all three domains, the asymmetry in question is one that involves the assignment of thematic roles. To capture this effect, we posit two distinct levels, or tiers, of cognition: non-grammatical cognition, more commonly associated with symmetric structures, and grammatical cognition more conducive to asymmetric structures. Within each of the three phenomenological domains, we find the distinction between non-grammatical and grammatical cognition to be manifest in three independent realms, phylogeny, ontogeny, and the architecture of human cognition. Thus, grammar constitutes the driving force behind the transition from symmetric to asymmetric cognitive structures.

## Introduction

This paper presents a preliminary and tentative formulation of a novel empirical generalization governing the relationship between grammar and cognition across a variety of independent domains.

Its point of departure is an abstract distinction between two kinds of cognitive structures: *symmetric* and *asymmetric*. A cognitive structure of the form XY is symmetric if X is to Y as Y is to X with respect to all relevant features. Conversely, XY is asymmetric if there is one or more relevant features applying differentially to X and Y, thereby effecting an ordering, ranking, or imbalance between X and Y.

While in principle any feature whatsoever has the potential for introducing asymmetry, this paper focuses on one specific feature, namely *thematic-role assignment*. Thematic roles are properties such as agent, patient, location, instrument and theme, that are assigned by one expression to another. For example, in a sentence such as *John ran*, the verb *ran* assigns the thematic role of agent to the noun-phrase *John*. Accordingly, due *inter alia* to thematic-role assignment, the sentence *John ran* is not a symmetric juxtaposition of its two words *John* and *ran*, but rather an asymmetric construction in which *ran* is a thematic-role assigner and *John* its thematic-role assignee.

Our main empirical finding concerns the role of language, or, more specifically, grammar, in effecting and maintaining the distinction between symmetric and asymmetric cognitive structures. Specifically, whereas symmetric structures devoid of thematic-role assignment more commonly occur in a non-grammatical and usually also non-verbal medium, asymmetric structures involving thematic-role assignment are more likely to be associated with a grammatical medium.

Our work draws together three independent strands of empirical research that we have been engaged in, separately and together, over the last several years, associated with three diverse phenomenological domains: *compositional semantics*, *metaphors* and *schematological hybrids*. Although quite different in many respects, these three domains share a common structural property, namely that they involve a bringing together of two or more subordinate entities into a single superordinate entity: X and Y become XY. For compositional semantics this consists of a juxtaposition of constituent signs to form a single more complex sign, e.g., *John* + *ran* > *John ran*; for metaphors this entails the bringing together of two different concepts in order to produce the comparison, e.g., *anger* + *volcano* > *Anger is like a volcano*; while for schematological hybrids this involves the combination of different entities to form a single new hybrid entity, e.g., *man* + *horse* > *centaur*. These three domains may thus be viewed as constituting conceptual combinations, in the sense of Murphy (1988, 1990), Wisniewski and Love (1998), and others.

As such, one may examine the extent to which the composite conceptual structures formed from the subordinate entities are symmetric or asymmetric in nature. For compositional semantics, the question is whether the meaning of, say, *John ran* is just the symmetric sum of the meanings of *John* and *ran*, or whether there are further asymmetries between *John* and *ran*, for example, as suggested above, the assignment by *ran* of the thematic role of agent to *John*. For metaphors, we examine the extent to which comparisons of two terms are symmetric and reversible, as in *Anger and a volcano are alike*, or alternatively asymmetric and irreversible, with a source term lower on a hierarchy of some kind, such as *volcano*, applying to a target term higher on the same hierarchy, such as *anger*. And for schematological hybrids, the issue is whether a centaur is merely a symmetric combination of half-man and half-horse, or whether it inherits more properties from one of its components than from the other, in accordance with various principles such as an Ontological Hierarchy, which might entail that the centaur would be more man than horse.

Our empirical results reveal a remarkable parallelism between the above three phenomenological domains. Within each domain, we find a strong tendency for symmetric structures to be associated with a non-verbal or otherwise non-grammatical medium, and a complementary preference for asymmetric structures to be associated with a grammatical medium. In other words, within each of the three domains, grammar introduces asymmetry. More specifically, we find that in all three domains, the asymmetry in question is one that involves, in some form or another, the assignment of thematic roles.

In order to capture this effect, we posit two distinct levels, or tiers, of cognition: *non-grammatical cognition*, more commonly associated with symmetric structures, and *grammatical cognition* more conducive to asymmetric structures. These two levels of cognition are not on a par; rather, grammatical cognition is derived from non-grammatical cognition by the introduction of thematic-role assignment, which has the effect of transforming symmetric structures into asymmetric ones.

Within each of the three phenomenological domains, we find the distinction between non-grammatical and grammatical cognition to be manifest in three independent realms. First, we show that the non-grammatical/grammatical distinction is a fundamental feature of the *architecture* of human cognition. Secondly, we demonstrate that the transition from non-grammatical to grammatical cognition is characteristic of *ontogeny*, the way cognition develops amongst infants. Thirdly, we offer indirect evidence and argumentation to the effect that a similar transformation from non-grammatical to grammatical cognition was also characteristic of *phylogeny*, the development of contemporary human cognition from that of our pre-human ancestors.

Empirical support for our findings derives from a mix of distinct research methodologies involving experimentation, observation of naturalistic behavior, and deductive argumentation within each of the three domains. Although we have already accumulated a large body of evidence in support of our findings, our presentation here is of a preliminary and programmatic nature, an initial laying out of the terrain to be filled in, hopefully, by future and more detailed studies.

In the next section we provide a brief characterization of the role of thematic-role assignment in effecting a distinction between symmetric and asymmetric structures, following which, in the subsequent three sections we survey the evidence for distinct non-grammatical and grammatical modes of cognition in compositional semantics, metaphors and schematological hybrids respectively. The section on Compositional Semantics represents work in progress by the first author, some preliminary results of which are presented in Gil (2007, 2008, 2015). The section on Metaphors represents work by the second author, some of which is reported on in Porat and Shen (2017) and Shen and Porat (2017). And the section on Schematological Hybrids represents joint collaborative work in progress by both authors, some of which is summarized in Shen and Gil (2017) and references therein.

## Thematic Role Assignment

Thematic roles are most familiar to us from linguistic theory. An important part of a word’s meaning is its associated thematic roles, also known as semantic frames (Fillmore, 1982, 1985 and others). For example, in order to understand the meaning of the word *hit*, one must know that it assigns its arguments two thematic roles: an agent and a patient.

Thematic role assignment is not specific to language; it is a feature of general conceptual structure reflecting our understanding of the world around us (Jackendoff, 1983, 1987, 1990). Thus, when we entertain the concept ‘hit,’ we know that it involves an agent and a patient, and when we attempt to identify the entities bearing these two roles, we engage in the assignment of thematic-roles at the level of conceptual structure. The independence of thematic-role assignment from language is evident from the behavior of animals, such as for example, great apes. As shown by de Waal (1982) and others, a chimpanzee observing one conspecific hitting another will infer that the one doing the hitting is more dominant on the social hierarchy than the one being hit: such an inference relies crucially on the distinction between thematic roles of agent and patient, and is obviously drawn without recourse to language.

The way in which thematic-role assignment effects asymmetric structures, illustrated with the sentence *John ran*, may be represented schematically as in (1) below:

(1)
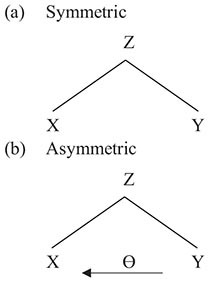

In (1a), X and Y combine to form a symmetric structure Z. In (1b), Y assigns a thematic role, denoted Θ, to X, thereby introducing an asymmetry to Z.

Although logically the distinction between symmetric and asymmetric structures is a clear cut binary one, in practice it is quantitative. Purely symmetric structures are hard to come by. Thematic roles aside, an otherwise symmetric structure will often exhibit a degree of asymmetry associated with the medium with which it is associated. For example, even in the otherwise symmetric (1a), X precedes Y in its orthographic representation on the page; in other cases an otherwise symmetric structure may exhibit an asymmetry, such as up vs. down, associated with the spatial medium.

A crucial characteristic of the distinction between symmetric and asymmetric structures is its *privative* nature. Asymmetric structures are derived from symmetric ones by adding features that effect the asymmetry. For example, in (1) above, the asymmetric structure in (b) is derived from its symmetric counterpart in (a) by introducing thematic-role assignment. Thus, symmetric structures are architectonically prior to asymmetric ones; they provide the foundations on which asymmetric structures are constructed.

As we shall demonstrate below, the processes by which asymmetric structures are built on top of symmetric ones are associated with the introduction of language. Although, as noted above, thematic-role assignment is part of general conceptual structure, it is through the medium of grammar that it assumes its role as a central feature underlying asymmetric cognitive structures, thereby providing the basis for the distinction between non-grammatical and grammatical levels of cognition.

## Compositional Semantics

The first of three phenomenological domains to be considered here is that of compositional semantics, which refers to the way in which the meaning of a combination of signs is derived from the meanings of each of its individual constituent signs.

**FIGURE 1:**
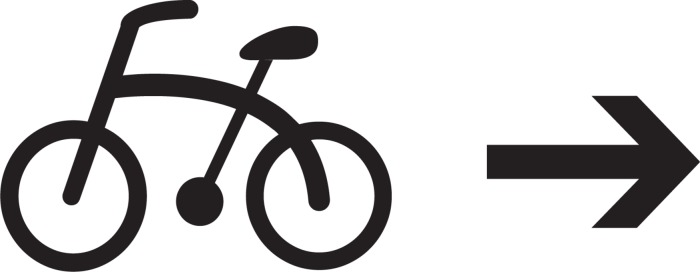
Compositional semantics: bicycle and arrow.

Since language is our primary conveyor of meanings, compositional semantics is most commonly thought of as a specifically linguistic feature; in fact, however, it is a central property of any semiotic system. Pictograms provide a fine illustration of this. Consider the juxtaposition of two meaning-bearing signs in Figure 1 below.

In Figure 1, the meanings of the individual signs can be paraphrased as ‘bicycle’ and ‘thataway’ respectively. But what do they mean in combination? In many European cities, similar combinations of signs are commonly used to mark bicycle lanes; however, they can also be used in other contexts, for example to point toward the location of a bicycle sale. Given such variation, one might suggest that juxtapositions such as that in Figure 1 are multiply ambiguous. Instead, as argued in Gil (2017), the combination of signs in Figure 1 has a single underspecified meaning which may be represented as follows:

(2) A (BICYCLE, THATAWAY)

In (2) above, the symbol A denotes the *association operator*. In its monadic form, the association operator corresponds in its interpretation to familiar genitive or possessive constructions; for example, A (JOHN) means ‘entity associated with John,’ or simply *John’s*, where the relationship between the associated entity and John is underspecified. For example, *John’s picture* could refer, depending on context, to the picture that John owns, the picture that John drew, the picture that portrays John, and so forth. However, in (2), the association operator appears in dyadic guise, where its meaning is ‘entity associated with bicycle and thataway.’ It thus provides an underspecified meaning encompassing all of the potential interpretations of Figure 1, that is to say, anything that has to do in some way with bicycle and thataway. In particular, it says nothing about the thematic role of *bicycle*, and, in particular, whether it is the theme (i.e., the thing that is going) or the goal (i.e., where you get to) of *thataway*.

The meaning represented in (2) is for all intents and purposes symmetric. Obviously, the two constituent meanings had to be written in some order on the page, but the order chosen is immaterial, the formula in (2) could just as easily have been written A (THATAWAY, BICYCLE) without any change in meaning. Thus, the combination of signs in Figure 1 and their single underspecified interpretation in terms of the formula in (2) provide a straightforward example of the symmetry characteristic of compositional semantics in a non-linguistic medium. Such interpretations, represented in terms of the polyadic association operator alone, may be referred to as *bare-associational* interpretations.

It is not by chance, however, that the formula in (2) has no easy translation into English and many other languages. In order to approach the meaning conveyed in (2) one needs to shed the straitjacket of grammar and construct a grammatically defective utterance with a telegraphic feel such as the following:

(3) Bicycles thataway

Constructions such as that in (3) are discussed in detail in Progovac (2015). Like (2), the interpretation of (3) is underspecified with regard to thematic roles. However, (3) is stretching English to its limit. A more natural rendition of (2) into grammatical and idiomatic English must necessarily choose between one of a number of more specific interpretations of (2) involving specific assignments of thematic roles to *bicycle*, such as the following:

(4)
  (a) Bicycles go thataway
  (b) Go thataway for bicycles

Building on the representations in (1), the most readily available interpretations of the two sentences in (4) may be represented as follows:

(5)
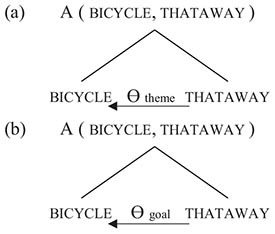

Whereas in (5a), *thataway* assigns the thematic role of theme to *bicycle*, in (5b) it assigns it the role of goal.

The contrast between (2) and (5) shows how grammar introduces asymmetry into semantic compositionality. Whereas (2), associated with the non-verbal pictogram in Figure 1, is symmetric, the two options in (5), corresponding to the English sentences in (4), are asymmetric, by dint of the asymmetric relationship of thematic-role assignment, in which THATAWAY assigns the appropriate thematic role to BICYCLE.

More specifically, the contrast between (2) and (5) shows how asymmetric structures are constructed on the foundations of symmetric ones. Note how the formula in (2), A (BICYCLE, THATAWAY), also forms part of the two representations in (5). This captures the central role that the polyadic association operator plays not just in pictograms but also in ordinary language. Imagine a person who does not know English but who has access to an English dictionary. It suffices for them to look up the meanings of the words *bicycle* and *thataway*, in order to be relatively certain that the meanings of both (4a) and (4b) have something to do with BICYCLE and THATAWAY, as specified by the association operator in the formula A (BICYCLE, THATAWAY). However, without knowledge of English grammar, they will have a harder time figuring out the difference in meaning between the two sentences in (4), and the details of thematic-role assignment distinguishing between them. Thus, the two formulas in (5) capture a fundamental feature of the architecture of compositional semantics, showing how the asymmetric grammatical process of thematic-role assignment, associated with the higher grammatical level of cognition, is built on top of the symmetric pre-linguistic structure effected by the polyadic association operator, associated with the lower non-grammatical level of cognition.

(It should be kept in mind that the asymmetry represented in (5) obtains between the assigner and the assignee of a single thematic role. This asymmetry underlies and sets the stage for another kind of asymmetry that has been the focus of much attention in recent linguistic literature, that which holds between two or more expressions in the same clause bearing different thematic roles; see, for example Kayne (1994) and Moro (2000). The symmetry under consideration here is thus logically prior to, and presupposed by, the latter and more commonly discussed notion of asymmetry.)

The architecture of compositional semantics expressed in the two formulas in (5) is mirrored by the transition from symmetric to asymmetric structures in ontogeny and phylogeny. Consider, first, early child language acquisition, where the child has just begun to produce two word utterances. Bloom (1973) cites the following examples from the spontaneous speech of 20-month-old Allison, who is playing with a pig inside a toy truck. The pig is hurt by a sharp corner of the truck, at which point Allison produces the following utterances:

(6) 
  (a) hurt truck *HURT - cause*
  (b) hurt knee *HURT - patient*

Given the context, in (6a) *truck* is understood as the cause of *hurt*, while in (6b), *knee* is understood as its patient — as indicated to the right. Accordingly, Bloom argues that there is no reason to analyze utterances such as these in terms of grammatical structure involving thematic-role assignment. Rather, the juxtaposition of words in early child language may be assigned a bare-associational meaning represented in terms of the polyadic association operator, such as, for (6a), A (HURT, TRUCK), ‘entity associated with hurt and with truck’ (Gil, 2017, p. 484). Thus, early child-language compositional semantics resembles that of pictograms, as illustrated in Figure 1 above. It is the symmetric foundation that forms the basis for the subsequent development of asymmetric thematic-role assignment in the adult language.

In this respect, ontogeny recapitulates phylogeny. Rudimentary symmetric compositional semantics would appear to be present in the natural communicative systems of primates in their natural habitat (Arnold and Zuberbühler, 2006, 2012; Schlenker et al., 2014 and others). A somewhat more productive compositional semantics would seem to be accessible to apes in captivity. Two well-known cases are those of the Kanzi, a bonobo using lexigrams (Greenfield and Savage-Rumbaugh, 1990), and Chantek, an orangutan using American Sign Language (Miles, 1990). Some examples of Kanzi’s spontaneous sign-language production are presented below:

(7) 
  (a) LIZ HIDE *agent - HIDE*
  (b) WATER HIDE *patient - HIDE*
  (c) HIDE AUSTIN *HIDE - agent*
  (d) HIDE PEANUT *HIDE - patient*

Example (7) above forms a mini-paradigm, represented schematically at right, in which HIDE is either preceded or followed by a participant, which, in accordance with the utterance’s context as provided by the authors, may, in either position, be understood as either the agent or the patient of HIDE. There is thus no evidence for any grammatical assignment of thematic roles in Kanzi’s use of lexigrams; rather, the relationship between the two signs is semantically underspecified. As in the pictograms in Figure 1, and also early child language in (6), the juxtaposition of lexigrams has a single bare-associational meaning, represented in terms of the polyadic association operator as, for (7a), A (LIZ, HIDE), ‘entity associated with Liz and with hiding’ (Gil, 2017, p. 482). Thus, the bonobo Kanzi’s use of lexigrams exhibits purely symmetric compositional semantics. Similar observations hold also for the orangutan Chantek’s usage of American Sign Language. Given that the common evolutionary ancestor of great apes such as bonobos and orangutans is shared also by humans, it may be concluded that this common ancestor also had symmetric compositional semantics in the form of the polyadic association operator, which then formed the basis for the subsequent development of asymmetric thematic-role assignment in human language. Thus, as shown above, the development from symmetric to asymmetric compositional semantics in both ontogeny and phylogeny underlies the architecture of compositional semantics, with the asymmetric polyadic association operator providing the foundation on which asymmetric thematic-role assignment then takes place.

The distinction between symmetry and asymmetry in the domain of compositional semantics is not categorical but rather gradated. Thematic-role assignment is not something that is either present or absent; instead, it can be present to various degrees, depending on a wide variety of factors, both grammatical and extra-linguistic. An extensive empirical exploration of some of these factors is conducted in an ongoing study, the *Association Experiment*. While some preliminary results of the Association Experiment are presented in Gil (2007, 2008, 2015, pp. 308, 321–322), most of its results have not yet been published.

In the experiment, speakers of different languages are asked to judge the truth conditions of sentences in their languages. Stimuli consist of written sentences, each accompanied by two pictures; speakers are asked which picture is correctly described by the sentence (they also have the options of choosing both pictures or neither). The experiment contains 32 stimuli measuring the relevance of thematic-role assignment to compositional semantics. The stimuli are controlled for a variety of factors, such as the nature of the activity (e.g., reversible vs. non-reversible), the type of the participants (e.g., animate vs. inanimate), and the participants’ spatial orientation in the pictures. For each language, at least 30 subjects are examined, all of lower socio-economic status, in order to control, as much as is practically possible, for effects due to lifestyle and education. The experiment has been conducted on 69 languages.

In (8) and (9) below, two examples of stimuli are shown for four selected languages: English, Japanese, Yali (a Trans-New-Guinea language of Papua, Indonesia) and Tikuna (a language isolate of the Amazon region of Colombia). In (8) and (9), speakers of the respective languages are asked to judge whether the given sentence is true in the situation depicted, and in an alternative picture not shown here. The percentages indicate the proportion of speakers who accepted each sentence as a true description of the picture, for the stimuli presented here together with other structurally similar picture-sentence pairs.

(8) Stimulus 1
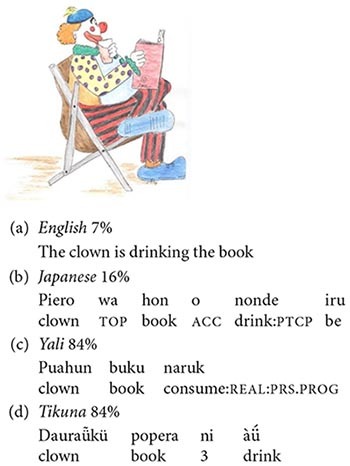

(9) Stimulus 2
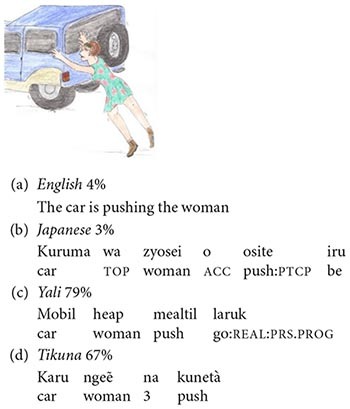

For each of the languages examined, the sentence in (8) is derived from a sentence such as ‘The clown is drinking the water’ by replacing the word for ‘water’ with the word for ‘book,’ while the sentence in (9) is derived from a sentence corresponding to ‘The woman is pushing the car’ by interchanging the words for ‘woman’ and ‘car.’

The Association Experiment measures the degree to which subjects distinguish between thematic roles by recourse to construction-specific rules of grammar involving morphosyntactic features such as word order and flagging (a cover term that includes case marking and adpositions). Consider, for example, English sentence (8a) *The clown is drinking the book*. In accordance with the polyadic association operator, the meaning of the sentence must have something to do with a clown, a drinking, and a book. And indeed, the test picture does involve a clown, a drinking, and a book. However, adult speakers of English overwhelmingly rejected sentence (8a) as a true description of the picture. This is because the compositional semantics of English contains much more than just the polyadic association operator: bare association is supplemented by thematic-role assignment. In particular, the structure of (8a) is such that *drink* assigns the thematic role of patient to *book*, which results in a semantically anomalous interpretation, while ruling out the test picture, in which *drink* and *book* are only loosely connected via bare association. Similarly, for English sentence (9a), *The car is pushing the woman*, the test picture does have a car, a pushing and a woman; however, adult speakers of English overwhelmingly rejected (9a) as a true description of the picture, because “it’s round the wrong way,” and the grammar is telling us, again anomalously, that the car is the agent of the pushing and the woman its patient.

The results of the Association Experiment provide further support for the two-tiered architecture of compositional semantics as represented in (5) and the way in which it plays out in ontogeny and phylogeny. Support for the two-tiered architecture in (5) is provided by a *wait-a-moment effect* produced by many subjects when responding to the experimental stimuli. For example, in (8), subjects would notice a clown drinking and a book and point to the picture, but then right after, realize that the grammar was wrong, say “wait a moment,” and retract their response and offer a negative one in its place. This effect points toward a two-stage process in which subjects first applied the symmetric polyadic association operator, as per (2), and only then, shortly after, added asymmetric thematic-role assignment, in accordance with (5). This two-stage process echoes Friederici’s (2002) neural model of sentence processing, and, in particular the “ELAN phase” occurring at 150–200 ms followed by the “LAN/N400 phase” at 300–500 ms.

Support for the ontogenetic trajectory from symmetry to asymmetry is provided by a study of children speaking the Riau dialect of Indonesian. While by age 10, subjects’ responses were at adult levels, 8 and 9 year old subjects were significantly more likely to ignore the adult-language preferences for particular thematic-role assignments and respond instead on the basis of bare association; for example, for (8) and (9), they would be more likely to point to the picture as being an acceptable interpretation of the corresponding sentence in Riau Indonesian.

Finally, support for the phylogenetic trajectory from symmetry to asymmetry is provided by inferences drawn from patterns of cross-linguistic variation in subjects’ responses to the experimental stimuli. Not all languages work the same way as English: as suggested by the percentages in (8) and (9), languages vary significantly in the degree to which bare association is narrowed down by additional grammatical rules governing the assignment of thematic roles. Whereas in languages such as English and Japanese, thematic-role assignment is largely specified by the grammar, and speakers usually reject bare-associational interpretations, in languages such as Yali and Tikuna, bare associational interpretations are obtainable in a majority of cases.

The degree to which thematic-role assignment is specified by the grammars of different languages is the product of several diverse factors, of which the most important one, which we focus on here, is the complexity of the polity with which the language is associated. It is no accident that many readers may not have heard of the two languages, Yali and Tikuna, chosen in (8) and (9) to exemplify greater tolerance of bare associational interpretations. The 69 languages of the Association Experiment sample may be ranked in accordance with a scale of polity complexity, as shown in (10) below:

(10) Polity Complexity
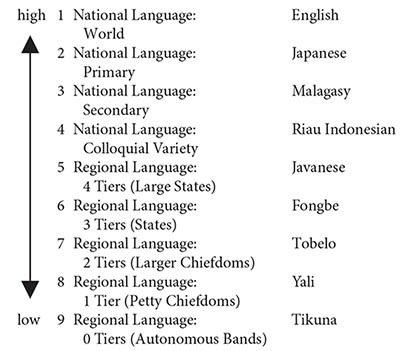

The scale in (10) combines several measures of polity complexity. First is a basic dichotomy between national and regional languages. National languages are further distinguished with respect to more specific characteristics pertaining to the language’s functions and status. And regional languages are classified in terms of the complexity of their associated societies as reflected in the number of levels of “jurisdictional hierarchy beyond local community,” as defined in the D-Place database (Kirby et al., 2016). In (10), each of the 9 levels of complexity is exemplified by one of the languages in the sample.

The 9-valued scale of polity complexity shown in (10) correlates positively with the degree of complexity of the compositional semantics of the associated languages, as evident in the results of the Association Experiment. In general, languages whose polities are of high complexity, such as English and Japanese, exhibit high grammaticalization of thematic roles and concomitant low tolerance of bare-associational interpretations, whereas languages of low polity complexity, such as Yali and Tikuna, exhibit low grammaticalization of thematic roles and high tolerance of bare-associational interpretations. Taking the 69 languages of the sample to be independent variables, the correlation turns out to be of high statistical significance. In the real world, though, the 69 languages are not all independent of each other; however, examining sets of closely related language varieties differing with respect to polity complexity provides even more convincing support for the correlation. For example, Standard Indonesian, with polity complexity 2, has higher grammaticalization of thematic-role assignment than Riau Indonesian, with polity complexity 4, which in turn has higher grammaticalization of thematic-role assignment than Minangkabau, with polity complexity 7 — even though all three language varieties are closely related exhibiting a certain degree of mutual intelligibility.

The correlation between polity complexity and grammaticalization of thematic-role assignment provides a direct window into the evolution of compositional semantics. Although we have no direct evidence with regard to the linguistic abilities of pre-modern humans or their hominin ancestors, we do know one obvious fact about their socio-political organization, namely that it was near the bottom of the scale of polity complexity in (10) above. Regardless of the nature and directionality of the causation underlying the correlation between polity complexity and grammaticalization of thematic-role assignment, the presence of the correlation suggests that the languages of today’s low-complexity polities may provide a model for the languages of our ancient ancestors: whatever today’s low-polity-complexity languages are like, that is how all languages used to be. The results of the Association Experiment thus provide further support for the conclusion that, in the course of the evolution of human language, compositional semantics began from bare association and the polyadic association operator, and gradually, over the course of time, evolved the grammatical structures that give rise to thematic-role assignment.

In summary, then, the Association Experiment provides additional evidence, architectural, ontogenetic and phylogenetic, for a two-tiered compositional semantics in which a symmetric polyadic association operator constitutes the foundation on which the asymmetric rules of thematic-role assignment may apply. In conjunction with the other sources of evidence discussed earlier, it thus shows how the asymmetry of thematic-role assignment is introduced by grammatical structure, both in the evolution of human language and in its acquisition by children — as is reflected in the two-tier architecture of compositional semantics represented in (5) above.

The results of this section thus run counter to many or most current approaches to compositional semantics in linguistic theory, in which asymmetric structures are posited directly, without recourse to a prior symmetric foundation. However, the two-tiered architecture argued for here would appear to be akin in spirit to Progovac’s (2015) approach, in which functional categories are built up on top of lexical ones, to form structures that also provide a reflection of an evolutionary past.

Compositional semantics represents one of the simplest and most ubiquitous domains in which two terms are brought together to form a third, and in which a pre-linguistic symmetric structure is rendered asymmetric by the introduction of grammar. We now go on to consider two additional phenomenological domains which also involve the bringing together of two terms, but which differ from compositional semantics in one important respect, namely that they involve some kind of conceptual anomaly.

## Metaphors

**FIGURE 2:**
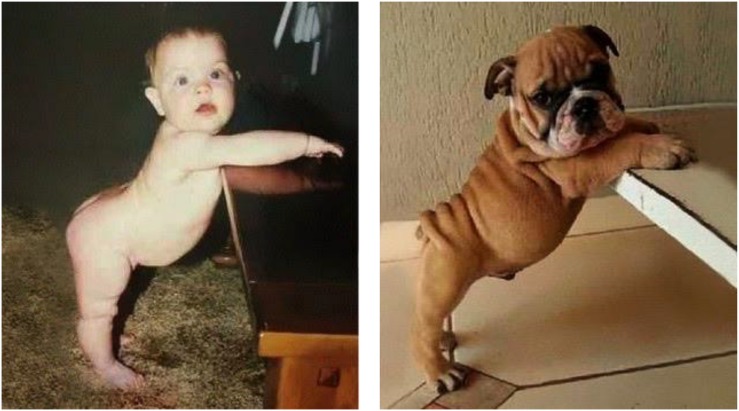
Metaphor: baby and dog.

Consider Figure 2 below, a popular internet meme, occurring under headings such as “funny lookalikes”.

In Figure 2, the baby and the dog assume near identical postures, resulting in two very similar spatial contours. The relation between the baby and the dog can be represented as in (11) below, where the symbol “∼” represents the relationship of similarity:

(11) BABY ∼ DOG

As represented in (11), the relationship of similarity between BABY and DOG is symmetric. From a purely logical point of view, if X is similar to Y then Y is similar to X. And indeed, in terms of processing, a search for similarities can just as readily start out by taking the baby as a reference point and seeking similar properties of the dog, or the other way around.

However, when people are asked to judge whether they prefer verbal comparisons in which the baby is said to resemble the dog, or alternatively ones in which the dog is said to resemble the baby, they exhibit a preference for the former. This preference is independent of the order in which the two entities are shown. (Indeed, the fact that the internet meme usually shows the baby to the left and the dog to the right, as in Figure 2, is probably a consequence of this preference, in conjunction with the predominance of left-to-right writing systems on the internet.) Experimental evidence for preferences such as these is provided in Connor and Kogan (1980) and Kogan et al. (1989).

Such preferences thus reveal an asymmetry, which may be represented as in (12) below, in terms of thematic-role assignment:

(12)
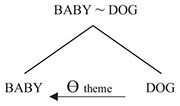

In (12), DOG assigns the thematic role of theme to BABY. (More specifically, as argued in Gil (2013), it assigns the thematic role of essant —a subrole of theme whose prototypical usage is in predicate nominal constructions such as *John is a teacher*.) What this says is that ‘baby is (like) a dog,’ where, of course, ‘is (like) a dog‘ is understood as something along the lines of ‘resembles a dog with respect to a particular set of properties.’ Conventional terminology captures this asymmetry by characterizing the dog as the *source* of the metaphor and the baby as its *target* (Lakoff and Johnson, 1980).

The contrast between the structures in (11) and (12) closely mirrors that between the structures in (2) and (5) in the preceding section. In both cases, thematic-role assignment imposes an asymmetry on an architecturally prior symmetric structure — the symmetry in question being that of bare association in the first case, similarity in the second. And as we shall see below, here too it is grammar that introduces the asymmetry in question.

Empirical evidence of various kinds has been offered in support of both bidirectional symmetric and unidirectional asymmetric approaches to metaphors. *Prima facie*, these different kinds of evidence appear to be contradictory. In reality, however, they reflect what Wolff and Gentner (2011) insightfully refer to as the “double life” of metaphors. And in fact, as shown in Porat and Shen (2017) and Shen and Porat (2017), the divergent conclusions are due to the variable mediums associated with the different sources of evidence. Specifically, while non-grammatical evidence lends support to bidirectional approaches, evidence based on grammatical phenomena tends to support unidirectional approaches.

Evidence for the bidirectional symmetric approach derives from various psychophysical experiments in which the manipulation of one domain affects the perception of another domain in ways that often correspond to hypothesized conceptual metaphors. For example, participants who held a warm (rather than cold) beverage in their hands tended to judge target individuals as having a warmer personality (Williams and Bargh, 2008), in accordance with the conceptual metaphor AFFECTION IS WARMTH; in another study, participants were likely to judge currency to be more valuable when they were holding a heavy (rather than a light) clipboard (Jostmann et al., 2009), in accordance with the conceptual metaphor IMPORTANT IS HEAVY. However, other experiments found effects applying in the direction opposite to that of the corresponding conceptual metaphor. For example, it was also found that manipulating participants’ feelings of social acceptance (by excluding or including them in a social game) can change their evaluation of room temperature — a mapping that defies the regular concrete-to-abstract pattern and has no verbal equivalent in ordinary language (Zhong and Leonardelli, 2008). Similarly, manuscripts that were evaluated as more important were experienced as heavier, in a reversal of the IMPORTANT IS HEAVY conceptual metaphor (Schneider et al., 2011). More generally, analysis of the various psychophysical experimental findings reveals a clear bidirectional pattern for many hypothesized conceptual mappings (see IJzerman and Koole, 2011 for an overview). Experiments such as these show that in the absence of an overt grammatical expression of the metaphor, the relationship between the two terms is bidirectional. Although such birectionality results from a combination of two opposing unidirectional processes, the cooccurrence of both processes means that, at a more abstract level, they “cancel each other out,” resulting in a pattern that may accordingly be characterized as symmetric (see Porat and Shen, 2017 and other articles in the same volume for further discussion).

The most common medium for the expression of comparisons is, however, verbal; and the linguistic nature of most experimental tasks is the reason why the bulk of the existing empirical evidence has always pointed toward a unidirectional, or asymmetric process. Thus, when the above-mentioned bidirectional experiential correlations are expressed in a verbal medium, the resulting metaphors are fundamentally unidirectional (Porat and Shen, 2017; Shen and Porat, 2017). For example, it is widely held (Lakoff and Johnson, 1980; Gibbs et al., 1994) that conventionalized metaphorical expressions such as *warm person* or *cold-hearted*, reflecting an underlying conceptual metaphor AFFECTION IS WARMTH, are cross-culturally unidirectional, in that they tend to map concrete domains, such as TEMPERATURE, on to abstract ones, such as INTERPERSONAL RELATIONS, rather than the other way around. Similarly, as noted previously, when confronted with stimuli such as those in Figure 2, speakers would rather say that the baby looks like the dog than the dog looks like the baby. Similar findings are reported in many other experimental and corpus studies (Connor and Kogan, 1980; Connor and Martin, 1982; Kogan et al., 1989).

It is sometimes suggested that the unidirectionality of metaphors reflects a conceptual asymmetry inherent to conceptual metaphors (Lakoff and Johnson, 1980). However, while this may be true in part, it cannot be the whole story; language, or more specifically grammar, plays a crucial role in introducing and amplifying the unidirectionality of metaphoricity. One obvious piece of evidence is provided by conceptually symmetric metaphors, in which the two terms are ontologically on a par, such as the following, adapted from Glucksberg and Keysar (1990):

(13) Surgeons and butchers are alike

(14) 
  (a) This surgeon is a butcher
  (b) This butcher is a surgeon

In (13), the two nouns occur in a symmetric coordination, whose most readily available interpretation is non-metaphorical (they both cut flesh). However, in (14), the two nouns occur in subject and predicate positions in a syntactically asymmetric predicate-nominal construction, with two significant consequences. To begin with, the asymmetric grammatical structure is itself conducive to metaphorical interpretations (Fishman, (n. d.)). Moreover, the metaphors expressed by the two sentences in (14) are in effect opposites: while in (14a) the surgeon is rough and careless, in (14b) the butcher is delicate and careful. Crucially, there is nothing in conceptual structure that can account for the different meanings of (14a) and (14b) and the opposite assignments of source and target in these two sentences. Rather, the different meanings that we associate with the two metaphors can only be attributed to the mirror-image syntactic structures in which they are expressed. In particular, it is the grammatical asymmetry of the predicate-nominal constructions in (14) that introduces the asymmetry of thematic-role assignment.

Thus, although forming the basis for competing theoretical approaches, bidirectionality and unidirectionality actually represent two distinct stages in the construction of metaphors, with symmetric comparisons such as those in (11) constituting the foundation for asymmetric metaphors such as those in (12), involving thematic-role assignment introduced by the grammatical medium.

Empirical support for a two-stage model for the comprehension of metaphorical comparisons is provided by Wolff and Gentner (2011). Subjects were asked to judge the comprehensibility of metaphors in either canonical order, e.g., *Some arguments are wars*, or reversed order, e.g., *Some wars are arguments*. When the metaphors were presented for a short duration of 500 ms, the sentences in the two orders did not differ in comprehensibility (although the metaphorical statements were still judged as being more comprehensible than nonsensical comparisons). In contrast, when the metaphors were presented for longer periods of time, the metaphors in canonical order were judged to be more comprehensible than their reversed counterparts. Their experiment thus provides direct evidence for a two-stage process in metaphor comprehension, with an earlier symmetric bidirectional stage followed by a subsequent later asymmetric unidirectional stage, again consistent with Friederici’s (2002) model mentioned earlier — though it says nothing about the role of grammar in this process.

Evidence for the role of grammar in the transition from bidirectionality to unidirectionality is provided by two further experiments conducted by Porat and Shen (2017) and Porat (in preparation). The first experiment made use of novel abstract-concrete concept pairs, such as childhood memories and migrating birds, while the second experiment made use of conventionalized concept pairs, such as fear and cold. Each experiment consisted of two phases.

In the first phase of each experiment, subjects were asked to arrange the pairs within the grammatically asymmetrical simile construction *__ is like __*. Subjects exhibited a strong preference for the concrete-to-abstract arrangement for both novel and conventional pairs, preferring sentences such as *Childhood memories are like migrating birds* and *Fear is like cold* over their reversed counterparts, *Migrating birds are like childhood memories* and *Cold is like fear*. This finding suggests that the conceptual asymmetry between the members of each pair was strong enough to dictate a preferred direction of mapping, regardless of the novelty/conventionality of the comparison.

In the second phase of each experiment, subjects were presented with the above pairs expressed either in a grammatically symmetric construction, e.g., *Childhood memories and migrating birds are alike*, or in a grammatically asymmetric construction e.g., *Childhood memories are like migrating birds.* For each item, subjects had to decide in which of two given contexts the sentence was more likely to be uttered: while one of the contexts was about the abstract concept, e.g., a nostalgic writer speaking about his youth, the other was about the concrete concept, e.g., an enthusiastic ornithologist describing the flight of birds. In this case, subjects preferred the context consistent with the concrete-to-abstract mapping, e.g., the nostalgic writer, not the enthusiastic ornithologist, significantly more often when presented with the grammatically asymmetrical construction than when presented with the grammatically symmetrical one, for both novel and conventional concept pairs. What this shows, then, is that despite the clear conceptual asymmetry between the two parts of the comparison, the abstract noun phrase, e.g., *childhood memories*, was not automatically assigned the role of metaphorical target; instead, this assignment occurred only after the two concepts were encountered in a grammatically asymmetrical structure.

The picture emerging from the above experiments is thus one of a two-tiered cognitive architecture, with a lower, non-grammatical level of cognition associated with symmetric bidirectional comparisons forming the basis for a higher level of cognition, in which asymmetric unidirectional metaphors are introduced and supported by the medium of grammar. Moreover, as was the case in the preceding section for compositional semantics, the two-tiered cognitive architecture of metaphorical comparisons can be shown to constitute a dual mirror of both ontogenetic and phylogenetic processes.

Several studies have shown that the unidirectionality of metaphors is a product of developmental maturation, and that for younger children bidirectionality is the rule. Connor and Martin (1982) applied the original task by Connor and Kogan (1980) to younger subjects and found that whereas the judgments of high-school students were similar to those of adult subjects, fifth- and seventh-graders exhibited no preference for a particular ordering of the test items. To see whether these findings were restricted to metaphors or reflect a general insensitivity to asymmetric comparisons, Connor (1983) investigated the judgments of third-, fifth- and seventh-graders, as well as college students, in a similar task involving asymmetrical literal comparisons. Again, while college students demonstrated significant inter-subjective agreement regarding the preferred order of each pair, this agreement decreased with age until it almost completely disappeared in the judgments of third-graders, for both metaphorical and literal comparisons. In a further study, Cerbin (1985) found that 4-years-olds are more likely to detect the metaphorical ground of grammatically asymmetrical comparisons, such as *A boat is like a leaf*, than grammatically symmetrical ones, such as *A boat and a leaf are alike*. In this respect, even pre-schoolers show some sensitivity to the difference between the two grammatical structures. However, the ordering of terms in the target and source roles did not affect the children’s performance: as in Conner’s study, a conventionally ordered sentence such as *A boat is like a leaf* was as easy to understand as its reversed version, *A leaf is like a boat*. Thus, as shown by these studies, metaphorical comparisons start out symmetric and bidirectional, and only later develop into their asymmetric unidirectional form.

A similar journey from bidirectionality to unidirectionality would appear also to be observable phylogenetically. In a cross-linguistic study, Gil et al. (in preparation) modify the Porat and Shen (2017) experiment above, presenting subjects with novel metaphorical constructions such as *A mackerel is like forgetfulness*, and asking them which of two potential speakers is more likely to utter the sentence — in the case at hand, a very old man or a fisherman. The experiment pits the directionality of conceptual hierarchies against the asymmetries of grammar, posing subjects with a dilemma. In accordance with the tendency to explicate abstract entities in terms of concrete ones, the comparison should be about forgetfulness, and hence the speaker is more likely to be the very old man. However, the grammatical structure of the sentence is such that the mackerel is the subject, and hence the speaker is more likely to be a fisherman. Who wins? Our findings, so far, suggest that the results depend on the language, and, in particular, on its associated polity complexity in accordance with the scale presented in the previous section in (10). Specifically, whereas in high-polity-complexity languages such as English, grammar wins out, with subjects exhibiting a strong preference to choose the fisherman as the speaker, in low-polity-complexity languages such as Abui (a language of the Timor-Alor-Pantar family of eastern Indonesia), grammatical and conceptual hierarchies are more equally balanced, with similar numbers of subjects choosing each of the two possible speakers. As was argued for compositional semantics in the preceding section, polity complexity may be used as a window into phylogeny, the assumption being that properties associated with languages of lower polity complexity are characteristic of a prior stage in the evolution of language and cognition. Specifically, we may conclude that at an earlier evolutionary stage, a somewhat weaker grammar played a relatively smaller role in the support of metaphor directionality.

Thus, the empirical evidence surveyed in this section shows that grammar plays a crucial role in the introduction of the asymmetry of thematic-role assignment into metaphorical structures — phylogenetically, ontogenetically, and in the cognitive architecture that mirrors these two developmental realms. Moreover, it does so in a way that presents a remarkable parallel to the way in which grammar was shown, in the preceding section, to introduce a similar asymmetry in a logically independent domain, that of compositional semantics. As we shall now see, a similar asymmetry-inducing role is played by grammar in yet a third, unrelated phenomenological domain, that of schematological hybrids.

## Schematological Hybrids

A *hybrid* is an entity conceptualized as an inseparable combination, or fusion, of components associated with two or more distinct entities, which may be referred to as the hybrid’s *parents*. The notion of hybrid is very broad; Wikipedia (accessed on 18 May 2016) offers links to 55 different entries with titles containing the term hybrid, concerned with a variety of items from domains such as biology (e.g., hybrid grape), technology (e.g., hybrid vehicle), art (e.g., hybrid genre), and many others (Shen and Gil, 2017, Gil and Shen, unpublished).

**FIGURE 3:**
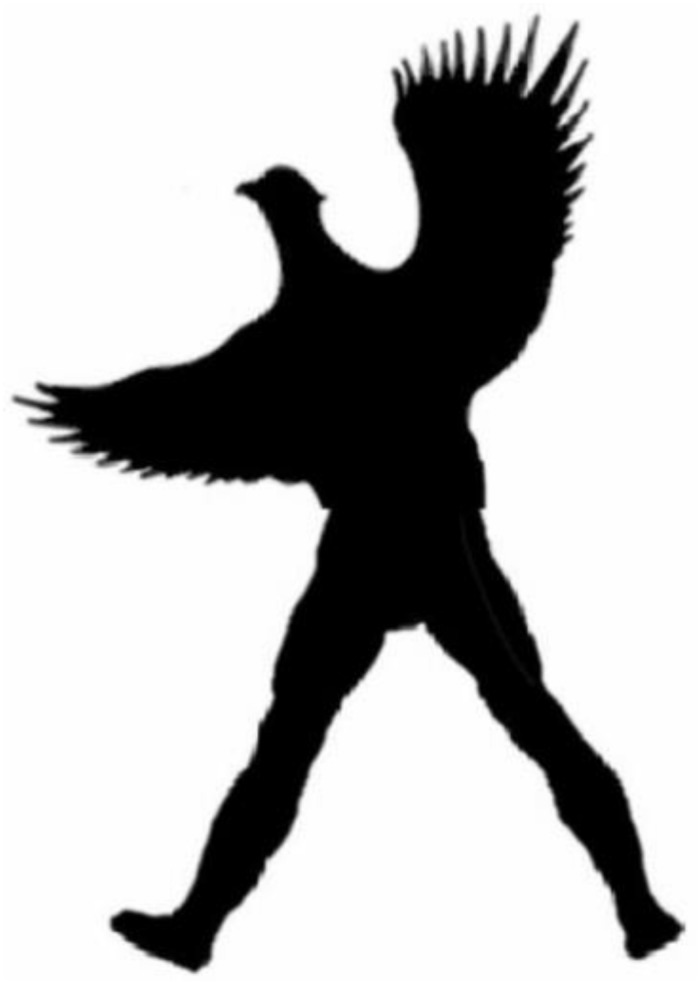
Schematological hybrid: man and bird.

An important subclass of hybrids is that of *schematological hybrids.* A schematological hybrid is one representable in a two- or three-dimensional image such as a statue or drawing. Some familiar examples of schematological hybrids include *centaurs*, part-human part-horse, and *mermaids*, combining the top half of a woman with the bottom half of a fish. Schematological hybrids are widespread in art, religion, folklore and popular culture, and have been around since time immemorial (see Wengrow, 2014, Gil and Shen, unpublished). The common occurrence of such hybrids in time and space suggests that they may reflect universal properties of human cognition. A novel example of a schematological hybrid is presented in Figure 3 to the right.

An important property of schematological hybrids is that while its parents are often familiar entities belonging to well-known categories, the hybrid itself is, or at least starts out as, a novel and unfamiliar entity whose categorial membership is not immediately obvious. For example, the hybrid in Figure 3 above clearly contains the top half of a bird and the bottom half of a man, but the entity as a whole does not instantiate any familiar concept, and there is no common conventional word for it: it’s just a “man-bird,” or something similar to that (Shen and Gil, 2017, p. 1179).

Schematological hybrids thus pose questions such as the following: What is it? What category does it belong to? For example, does the man-bird belong to the category of humans, or of animals? What properties does it have? For example, can the man-bird speak, or can it fly? More generally, one may ask whether one of the hybrid’s parents is more central to its conceptualization, and if so, which one? For example, is the hybrid in Figure 3 more man or more bird? In other words: is the construction of the hybrid conceptualized as symmetric or asymmetric?

In our work, we examone the ways in which the conceptualization of hybrids is governed by the *Ontological Hierarchy* (Keil, 1979; Connor and Kogan, 1980; Deane, 1992 and others), a basic knowledge structure that imposes a hierarchical order on different kinds of entities:

(15) The Ontological Hierarchy
  *humans* > *animals* > *plants* > *inanimates*.

Our focus is on the following question: What is the effect of the Ontological Hierarchy on the conceptualization of hybrids? Specifically, to what extent is there a tendency for hybrids to be categorized in accordance with the parent that is higher on the Ontological Hierarchy; for example, a man-bird as a kind of man, not as a kind of bird (Shen and Gil, 2017)?

Our main finding is that the Ontological Hierarchy is in fact relevant to the conceptualization of hybrids. However, the Ontological-Hierarchy effect depends crucially on the medium in which the categorization takes place; specifically, it is dependent on the presence of grammar. In the absence of grammar, subjects tend to conceptualize hybrids symmetrically; for example, a man-bird is not more manlike than birdlike, and when forced to choose, similar numbers of subjects will choose either option. However, in grammatical contexts they are more likely to verbalize the same hybrids asymmetrically, in accordance with the Ontological Hierarchy; for example. A man-bird might be described as a man with bird’s wings rather than a bird with man’s legs.

The effect of grammar on the categorization of hybrids may be observed in the following three ways (Shen and Gil, 2017, p. 1181):

(16) 
  (a) The Ontological-Hierarchy effect is greater for tasks that involve grammatical structure than for non-verbal tasks or tasks that involve just the lexicon.
  (b) The Ontological-Hierarchy effect is greater for non-verbal tasks when they are primed by verbal ones.
  (c) The Ontological-Hierarchy effect is greater for verbal tasks when there is “more grammar”; specifically, asymmetric vs. symmetric grammatical structures.

Our experimental studies make use of a set of 24 custom-designed visual stimuli representing schematological hybrids, such as that shown in Figure 3 above. The 24 hybrids instantiate all 6 possible binary combinations of the 4 categories of the Ontological Hierarchy: human–animal, human–plant, human–inanimate, animal–plant, animal–inanimate, and plant–inanimate. Each of these 6 combinations is represented by 4 stimuli, 2 in which the parent higher on the Ontological Hierarchy is located above the parent lower on the hierarchy, and 2 in which the parent higher on the Ontological Hierarchy is positioned beneath the other parent — as happens to be the case in Figure 3. This was in order to neutralize potential effects of spatial orientation on the hybrids’ categorization (Shen and Gil, 2017, p. 1183).

The first series of tasks examined the conceptualization of the hybrid stimuli in non-verbal and other contexts devoid of grammar. In the first *non-verbal categorization task* (Shen and Gil, 2013; reported on in Shen and Gil, 2017, p. 1185), Hebrew-speaking subjects were presented with the 24 hybrids; under each hybrid were two sets of visual images representing members of the two categories associated with each of the hybrid’s two parents. For example, for the man-bird hybrid in Figure 3, subjects were shown a set of images of humans and a set of images of birds. Subjects were asked to decide which of the two sets the hybrid belonged to. The results were around 50%, that is to say, at chance level.

In a similar *lexical label categorization task* (Shen and Gil, 2013; reported on in Shen and Gil, 2017, p. 1187), Hebrew-speaking subjects were presented with the same 24 visual hybrids; however, instead of being asked to assign the hybrid to a set of visual images, they were asked to match it with a descriptive word label. For example, for the hybrid in Figure 3, subjects were shown the word *iš* ‘man’ and the word *cipor* ‘bird.’ Although this task was verbal, it did not involve any recourse to grammar. And just like the previous task, the results were around 50% — at chance level.

In a somewhat different *color inference task* (Mansour, 2008; Shen and Gil, 2013; reported on in Shen and Gil, 2017, p. 1187), speakers of Arabic were shown visual images of the hybrid’s parents, each in a different color. Beneath the two parent images they were given a colorless silhouette of the appropriate hybrid. Subjects were then requested to infer the color of the hybrid based on the colors of its two parents. For example, for the hybrid in Figure 3, they might have been given a green man and a red bird: would the hybrid silhouette then be green or red? Again, subjects’ choices were at chance, as in the two preceding tasks.

Thus, the above series of tasks all show that in the absence of grammar, conceptualization of hybrids is symmetric: subjects are no more likely to categorize a visual stimulus of a hybrid in accordance with one of its parents than in accordance with the other. However, when grammar is introduced, an entirely different picture emerges, as is shown in the second series of tasks.

In the first *description task*, speakers of Hebrew were asked to produce a short verbal description of each of the 24 hybrids (Shen et al., 2006; Shen and Gil, 2013; reported on in Shen and Gil, 2017, p. 1183–1184). Their responses were then coded according to whether the description represented a conceptualization of the hybrid as (i) belonging to the category of the parent higher on the Ontological Hierarchy, (ii) belonging to the category of the parent lower on the Ontological Hierarchy, or (iii) neutral, not belonging to either category to the exclusion of the other. Examples of subjects’ responses to the stimulus in Figure 3 illustrating these three possibilities are provided in (17) – (19) below:

(17) Consistent with Hierarchy
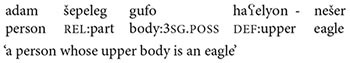

(18) Inconsistent with Hierarchy
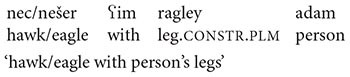

(19) Neutral
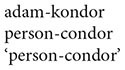

A large majority of the descriptions offered were asymmetric, as in (17) and (18); amongst these, roughly two-thirds of the descriptions were consistent with the Ontological Hierarchy, as in (17). (The remaining neutral descriptions, as in (19), were too few for any tendencies to be observed.)

In a second *choice of description task* (reported on in Shen and Gil, 2017, pp. 1186–1187), Hebrew-speaking subjects were shown hybrids from the basic set of 24 stimuli, where alongside each hybrid two descriptions were presented, one consistent with the Ontological Hierarchy, e.g., ‘man with bird’s wings,’ the other inconsistent with the hierarchy, e.g., ‘bird with man’s legs’. The results of this perception task mirrored those of the preceding task: subjects displayed a significant preference for descriptions in accordance with the Ontological Hierarchy.

In a third *choice of metaphor task* (also reported on in Shen and Gil, 2017, pp. 1186–1187), speakers of Arabic were shown the same hybrids, except that this time, each hybrid was accompanied by two metaphors based on the hybrid’s parents, one consistent with the hierarchy, e.g., ‘the man is like a bird,’ the other inconsistent with it, e.g., ‘the bird is like a man.’ Once again, subjects displayed a significant preference for metaphors that were constructed in accordance with the Ontological Hierarchy.

The contrast between the two sets of tasks is thus striking. While the first, non-grammatical set of tasks reveals a symmetric state of affairs in which neither of the hybrid’s parents is preferred over the other, in the second, grammatical set of tasks, grammar brings about a preference for hybrids to be categorized in accordance with the parent that is higher on the Ontological Hierarchy. In other words, grammar introduces asymmetric cognitive structures.

The effect of grammar on the conceptualization of hybrids is represented schematically in (20) and (21) below:

(20)
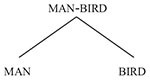

(21)
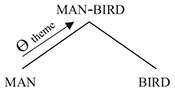

Whereas the structure in (20), representing the non-grammatical tasks, is symmetric, that in (21), representing the grammatical tasks, is asymmetric. As is the case for compositional semantics and metaphors previously, the asymmetry introduced by grammar involves thematic-role assignment — but in a rather different way. Whereas in (5) and (12) one of the two constituent terms assigns a thematic role to the other one, for hybrids, as represented in (21) above, the constituent term assigns a thematic role to the superordinate constituent. Specifically, the hybrid MAN-BIRD, as a whole, is assigned the role of theme by its parent MAN: the man/bird hybrid *is* a man. This specific configuration of thematic-role assignment may be viewed as a particular case of headedness, in which a property of the head constituent MAN percolates upwards to the superordinate constituent (Gil, 1985).

To this point, all of the tasks described involved speakers of Hebrew or closely related Arabic. However, given that what is at issue is an effect of grammar on cognition, it is reasonable to ask whether all languages work the same way as Hebrew and Arabic; after all, as is well known, although the Animacy Hierarchy itself is universal, its manifestations vary greatly from one language to another. To examine the cross-linguistic applicability of the animacy effect on the categorization of hybrids, we replicated two of the preceding tasks, the non-verbal categorization task and the verbal description task, in three additional languages: Bulgarian (Admon, 2008), Indonesian, and Minangkabau (Shen and Gil, 2013). In all three languages, the same pattern as in Hebrew was obtained: whereas in the non-verbal categorization task, categorization was roughly at chance, in the verbal description task, a significant Ontological-Hierarchy effect was in evidence.

So far, all of the tasks described here were off-line, dealing with the products of hybrid conceptualization. One may ask whether a greater Ontological-Hierarchy effect for tasks involving grammar is present also in the on-line processes of hybrid comprehension. To address this question we developed two *reaction-time tasks* (Mashal et al., 2014), summarized in Shen and Gil (2017). Both tasks showed that for the categorization of hybrids, the greater Ontological-Hierarchy effect associated with grammatical tasks in the off-line products of conceptualization is matched by a similar linguistic effect also in the on-line processes of hybrid comprehension.

A further *grammatical priming task* provides evidence for a rather more striking version of the effect of grammar, namely that, as formulated in (16b), the Ontological-Hierarchy effect is greater for non-verbal tasks if they are primed by verbal ones (Shen and Gil, 2013; reported on in Shen and Gil, 2017, 1194–1195). The verbal priming task sequence was performed in two stages 1 week apart. In the first stage, speakers of Hebrew performed the non-verbal categorization task. In the second stage, the same subjects were requested to perform the non-verbal categorization task again; however, before categorizing each hybrid, they were asked to produce a verbal description. The results showed that hybrids would be more likely to be non-verbally categorized in accordance with the Ontological Hierarchy if such categorization took place right after the grammatical description task.

To this point, we presented a variety of experimental studies showing that, in accordance with (16a) and (16b), the Ontological-Hierarchy effect is greater for tasks that involve, or are primed by, grammatical structure, than it is for non-verbal tasks or tasks that involve only the lexicon. One may now ask whether it is the mere presence of grammar that is responsible for the observed hierarchy effects, or conversely whether some specific feature of grammatical structure might underlie the role of the Animacy Hierarchy in the categorization of hybrids. Two further studies point toward the latter alternative. Specifically, they suggest that the crucial property of grammar responsible for the hierarchy effects is the pervasive asymmetry that is characteristic of most grammatical constructions: as specified in (16c), more grammatical asymmetry leads to more of an Ontological-Hierarchy effect (reported on in Shen and Gil, 2017, pp. 1191–1194).

Consider, for example, a garden-variety verbal description of the hybrid in Figure 3: *man with bird’s head*. The two nouns denoting the hybrid’s two parents, *man* and *bird*, are not of equal status; rather, they embody an array of grammatical asymmetries, pertaining to features such as linear order, c-command, agreement, and semantic referentiality. Grammatical asymmetries such as these present a natural target for the Ontological Hierarchy to map on to, in the variegated ways that linguists generally subsume under the workings of the Animacy Hierarchy.

Consider, now, an alternative description of the hybrid in Figure 3, involving a coordination: *man and bird*. In contrast to the previous example, *man and bird* displays just one asymmetry, that of linear order: *man* occurs before *bird*. We shall thus refer, somewhat loosely, to coordinative constructions as symmetric, in contrast to other constructions which exhibit a larger variety of grammatical asymmetries. Alternatively, one might say that asymmetric constructions exhibit “more” grammar than their (almost) symmetric coordinating counterparts.

As specified in (16c), the Ontological-Hierarchy effect on the conceptualization of hybrids is more pronounced for verbal tasks when there is “more grammar,” involving asymmetric structures, than it is when there is “less grammar,” as is the case for symmetric structures. Evidence comes from the measurement of reaction times, as in the tasks discussed above (Mashal et al., 2014). Speakers of Hebrew were shown schematological hybrids and potential verbal descriptions, and asked to judge whether each description was appropriate for the corresponding hybrid. The verbal descriptions were of the following kinds: (a) asymmetric descriptions, either in accordance with the Animacy Hierarchy, as in (17), or in opposition to it, as in (18), or (b) symmetric descriptions, as in (19), in which the order of the two items was consistent or inconsistent with the hierarchy.

If the hierarchy effect shown previously is due solely to the verbal medium and the presence of grammatical structure, then we might expect to observe differences in reaction time between the two cases: (a) for asymmetric descriptions, shorter reaction times for descriptions in accordance with the Animacy Hierarchy than for descriptions in opposition to it, and (b) for symmetric descriptions, shorter reaction times for descriptions in which the order of the two items was consistent with the hierarchy than for descriptions in which the order of the two items was inconsistent with it. On the other hand, if the hierarchy effect is dependent specifically on the presence of grammatical asymmetries, then one would expect to observe reaction-time differences only in the former (a) case, with the asymmetric descriptions, but not in the latter (b) case, with the symmetric descriptions. And in fact, this is what the results of the experiment showed: reaction-time differences were observed for the asymmetric descriptions but not the symmetric ones (reported on inShen and Gil, 2017, p. 1193).

Thus, the online judgment task reveals that it is not the grammatical medium itself but rather the presence of asymmetric grammatical structures that introduces the Animacy-Hierarchy effect. In accordance with (16c), then, more grammar means more of an Animacy-Hierarchy effect in the categorization of hybrids.

We have thus provided empirical evidence for three distinct but related ways in which grammar introduces asymmetries in the conceptualization of hybrids, as spelled out in (16a–c). As was the case in the preceding sections, for compositional semantics and metaphors, the two-tiered cognitive architecture of hybrid conceptualization can now be shown to constitute a reflection of both ontogenetic and phylogenetic trajectories.

While 10 and 6 years old speakers of Hebrew were found to perform at adult level with respect to the non-verbal categorization task and description task (Aleluf, 2005), some significant differences emerged when the same two tasks were performed by 3 years olds (Sanhedrai, 2017). As pointed out earlier, in the case of the description task, most of the descriptions offered by adults were asymmetric — either in accordance with the Ontological Hierarchy, as in (17), or, in smaller numbers, in violation of it, as in (18). However, for the 3 years old, a significantly larger number of descriptions offered were symmetric, as in (19). Thus, children follow an ontogenetic trajectory mirroring the two-tiered architecture of hybrid conceptualization observed amongst adults. Specifically, just as the asymmetric non-grammatical mode of hybrid categorization forms the foundation upon which the symmetric grammatical mode is constructed, so younger infants start out with more symmetric descriptions of hybrids, before moving on to more asymmetric descriptions as they mature.

An additional manifestation of the same ontogenetic path from symmetric to asymmetric categorization of hybrids becomes evident in a more fine-grained analysis of the performance of the 3-year-old children. Like with the older groups, the hierarchy effect was significantly higher for the description task than for the non-verbal categorization task. However, for both tasks, the hierarchy effect was weaker overall than it was for the older groups; see Shen and Gil (2017) for additional details. These facts thus provide further support for the presence of an ontogenetic trajectory from symmetric to asymmetric conceptualization of hybrids, one that mirrors the two-tiered architecture of hybrid conceptualization amongst adults.

One may now ask whether here, too, in the domain of hybrid conceptualization, ontogeny also recapitulates phylogeny. Given the lack of archeological attestations of schematological hybrids amongst hominins, and the obvious challenges posed by conducting experiments involving hybrids on primates, direct evidence is hardly forthcoming. Still, we do know that higher animals are clearly capable of non-verbal categorization (Zentall et al., 2008); and we know that they don’t have grammar. On this basis, it would seem plausible to assume, as a default hypothesis, that their categorization of hybrids would resemble that of humans in a non-grammatical mode, that is to say, it would be symmetric.

Some preliminary indirect support for this assumption is provided by a hybrid description task performed by native speakers of Arabic, in two different registers, standard and colloquial (Kadan, 2019). The task was designed to test for possible effects of the medium in which the description is couched. Whereas in the previous description tasks the descriptions were written, in the present study written descriptions were compared with oral ones. For both standard and colloquial registers, the written descriptions were in accordance with the Ontological Hierarchy, replicating their counterparts in Hebrew and other languages. However, the oral descriptions did not exhibit an Ontological-Hierarchy effect. Since writing is a relatively recent innovation in human history, one may tentatively conclude that differential cognitive behavioral patterns associated with oral and written language may reflect earlier and later points on an evolutionary trajectory. In the case at hand, then, the symmetric descriptions of the oral task would represent an earlier evolutionary stage than the asymmetric descriptions of the written task, thereby suggesting that for schematological hybrids as well, phylogeny also embraces a journey from symmetry to asymmetry.

## Conclusion

The empirical findings presented in this paper demonstrate a striking and hitherto unobserved parallel between three quite different phenomenological domains of human cognition, pointing toward a central role played by grammar in the architecture, ontogeny and phylogeny of cognition. These findings are summarized in Table 1.

**TABLE 1:** Symmetry and asymmetry in compositional semantics, metaphors and schematological hybrids.

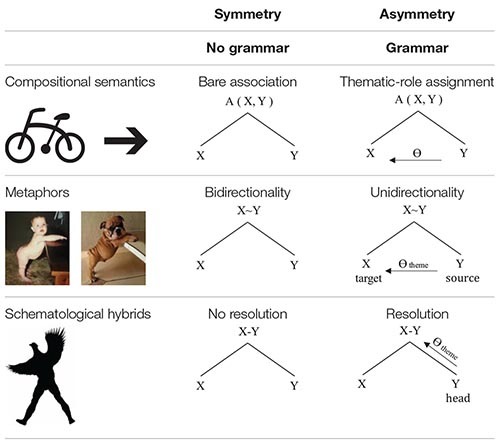

In Table 1, the three rows represent the three phenomenological domains discussed in the preceding three sections illustrated, in the first column, with their respective leading examples reproduced from Figures 1–3 respectively. The remaining two columns, recapitulating the structures posited in (1) and (5), (11) and (12), and (20) and (21), show the symmetric structures associated with the absence of grammar, contrasted with the asymmetric structures resulting from the introduction of grammar.

In all three domains, the asymmetry introduced by grammar involves thematic-role assignment, albeit in rather different configurations. Whereas for compositional semantics and metaphors, one of the two terms assigns a thematic role to the other, for schematological hybrids, the term in question assigns a thematic role to the superordinate term, pointing toward its characterization as the head of the construction. Moreover, whereas for compositional semantics, it is thematic-role assignment *per se* that is introduced by grammar, in the two remaining domains, thematic-role assignment is put to service to effect a further asymmetry: for metaphors, their unidirectionality and the distinction between source and target terms, and for schematological hybrids, their resolution and identification with one of their parents to the exclusion of the other. Finally, whereas for compositional semantics, any thematic role may be involved, in the case of metaphors and schematological hybrids, the thematic role involved is that of theme.

The role of theme made reference to in this paper is somewhat broader than that which is commonly assumed within many grammatical theories, which tend to focus on more semantically specific roles such as agent, patient, source, locative and so forth. To say that B assigns the role of theme to A is to assert that B *applies to* A, or in other words that B describes, characterizes or says something about A. Within some variants of formal semantic theory (Keenan, 1979; Barwise and Cooper, 1981; Keenan and Faltz, 1985), the theme A is an *argument*, while the thematic-role-assigner B is its *function*. Alternatively, within the more psycholinguistically oriented theory of conceptual combination (Rumelhardt, 1980; Cohen and Murphy, 1984; Murphy, 1988, 1990), the theme A is associated with a *schema*, while the thematic-role-assigner B *fills a particular slot* within that schema. It should be kept in mind that the relationship between A and B is not one of predication in the usual sense of the word; while in some cases B may indeed be predicated of A, in other cases B may stand in an attributive relationship to A. Similarly, the relationship between A and B is not a pragmatically based relationship such as topic-comment; whereas in many contexts A may be the topic and B its comment, in other contexts a variety of other discourse configurations may obtain. Instead, we view thematic roles, including *inter alia* the generalized role of theme, as constituting particular manifestations of a deeper and more fundamental asymmetric semantic relationship integrating properties of the argument/function relation of formal semantics and the schema/slot-filler relation of conceptual combination theory.

Why should grammar introduce asymmetric thematic-role assignment into otherwise symmetric cognitive structures? Given that thematic roles are part and parcel of our general conceptual structures, it is not obvious why their occurrence, in domains as diverse as compositional semantics, metaphors and schematological hybrids, should require, or at least be strongly supported by, the presence of grammar. We speculate that the answer to this question may lie in the central role played by the twin relations of predication and attribution in grammatical organization. In Gil (2012) it is argued that predication and attribution are composite emergent structures resulting from the conventionalized convergence of thematic-role assignment and headedness. Specifically, a predicate is defined as a thematic-role-assigner head while its arguments are its thematic-role-bearing modifiers; conversely, an attribute is defined as a thematic-role-assigner modifier while its head is its thematic role-bearing head. Like thematic-role assignment, as pointed out earlier, headedness is also an element of general conceptual structure, manifest in diverse domains ranging from our conceptualization of every-day objects through tonal music to language, and, within language, from phonology through syntax to discourse structure — see Gil (1985) and references therein. However, unlike thematic-role assignment, we are, at present, unaware of any evidence to the effect that headedness is present in the cognitive structures of non-human animals. Whereas thematic-role assignment and headedness are part of general conceptual structure, their convergence in the form of predication and attribution is thus specific to grammar. We conjecture that it is the pervasive nature of predication and attribution in grammar that is responsible for the introduction, through grammar, of thematic-role assignment into cognitive structures such as those associated with compositional semantics, metaphors and schematological hybrids.

To summarize, this paper has provided novel empirical evidence, from compositional semantics, metaphors and schematological hybrids, for the existence of two distinct levels, or tiers of cognition, non-grammatical and grammatical, the latter derived from the former by the introduction of thematic-role assignment and its associated asymmetries. This two-tiered architecture, with grammatical cognition placed on top of non-grammatical cognition, is argued to reflect the phylogeny and ontogeny of cognition, proceeding hand in hand with the evolution and development of language and grammar.

The results of this paper may perhaps be construed as supporting a variant of the so-called Whorf Hypothesis, one in which it is not the distinctive properties of particular languages, in contrast to other ones, that differentially shape our thought processes, but rather the universal properties shared by all languages that affect our common processes of conceptualization. This would also be in line with Slobin’s (1996) notion of “speaking for thinking,” where the act of representing the conceptualization of non-verbal stimuli in language leads to the rendering of such conceptualizations into the grammatical structures made available by the language, resulting in the subsequent adaption and modification of the conceptualizations in accordance with these grammatical structures.

As important as we consider them to be, the findings of this paper remain tentative and preliminary. We expect that future investigations into other phenomenological domains will reveal further instances of grammar introducing asymmetries into cognitive structures, thereby providing further support for the distinction between non-grammatical and grammatical cognition, and, *ipso facto*, for the central role that grammar plays in human cognition.

## Data Availability Statement

The datasets generated for this study are available on request to the corresponding author.

## Ethics Statement

Ethical review and approval was not required for the study on human participants in accordance with the local legislation and institutional requirements. Written informed consent from the participants’ legal guardian/next of kin was not required to participate in this study in accordance with the national legislation and the institutional requirements.

## Author Contributions

DG and YS contributed equally to all aspects of this manuscript. The order in which the authors are listed is merely alphabetical.

## Conflict of Interest

The authors declare that the research was conducted in the absence of any commercial or financial relationships that could be construed as a potential conflict of interest.
